# Multisensory integration in insect flight control

**DOI:** 10.1098/rsbl.2023.0565

**Published:** 2024-01-24

**Authors:** Sanjay P. Sane, Ric Wehling, Tom Daniel

**Affiliations:** ^1^ Tata Institute of Fundamental Research, National Centre for Biological Sciences, Bengaluru, Karnataka 560065, India; ^2^ Air Force Research Lab, Eglin, FL 32542, USA; ^3^ Department of Biology, University of Washington, Seattle, WA 98195, USA

**Keywords:** feeding behaviour, gaze stabilization, aggregation in social caterpillars, local specialization in insect eyes, integration of vision and olfaction

## 1. Introduction

Animals face the challenge of discriminating between the sensory consequences of their own motor actions, as opposed to sensory stimuli resulting from events taking place in the external world [1]. Indeed, this ability is key to nearly all locomotor behaviours such as territorial chases, prey-tracking or positional control, and serves as an inspiration for the development of artificial autonomous vehicles. Insects are especially fascinating from this perspective because they accomplish these tasks in flight, at extraordinary speeds, yet with incredibly stringent size, weight and power requirements (e.g. [2]). This suggests that they are able to extract, filter and process their sensory feedback in ways that we do not fully comprehend.

Traditionally, researchers have assumed that insect behaviour is determined by external stimuli which trigger a chain of activities ranging from sensory signal transduction to the generation of motor commands [3]. This includes the parallel acquisition and processing of information within a given sensory system followed by cross-modal signal integration to generate robust multimodal feedback signals in pre-motor and motor circuits, ultimately causing muscle contraction and movement. In sharp contrast to this notion, animal nervous systems can internally generate motor activity, in the absence of external stimuli (e.g. [4]).

This broad theme is explored by five papers in this special mini-issue centred on the topic of processing of multisensory information in insects. These studies, which focus on diverse insect systems, offer unique insights into how insect nervous systems acquire, process, integrate and weigh information from disparate sensory modalities.

In the first paper, Ruiz & Theobald [5] explore how multisensory integration enables corrections to disturbances in the flight trajectory of flies. They point out that, whereas rapid flight trajectory corrections in response to perturbations are strongly mediated by mechanosensory feedback (e.g. halteres in flies), the role of visual inputs is pre-eminent over longer timescales. Specifically, they explore which features of the visual world are most critical for in-flight path correction. Visual and mechanosensory modalities are widely recognized as two complementary and critical sensory modalities for locomotor control, yet the nature of the critical features of the visual world, particularly in natural environments, represents an open problem. For example, the image statistics of natural environments differ considerably from the simple visual sensory stimuli that are typically used in studying visual motion sensing. In natural environments, the image statistics of scenes above the fly (the dorsal field of view) differ considerably from that beneath the flying animal—sky views present simpler moving images than ground views. The authors presented laterally moving dot fields of different densities (above and below the tethered fly) and found that, in the fly *Drosophila melanogaster*, the amplitude of stabilizing responses depends strongly on the density of dots. The dorsal field of view has little effect, whereas the ventral field strongly modulates counter-steering. This paper thus shows that both vision and mechanosensory systems are important, but local specialization of the eye in the lower region also plays a critical role in translational flight stabilization.

The second paper, by Deora *et al*. [6], probes the role of both mechanosensory and visual sensory systems and their influence on the feeding behaviour of the tobacco hornworm hawkmoth *Manduca sexta*. These moths feed while hovering above a flower and probe the surface of the flower with their long flexible proboscis to localize a tiny nectary opening in which they insert their proboscis to feed. As this task is strongly tactile, mechanosensory input is critical [7]. However, the precise role of visual input in this behaviour, although essential, is somewhat unclear. To address this question, the authors quantified hovering flight patterns and feeding performance of freely behaving hawkmoths feeding from three-dimensional printed artificial flowers under two different light conditions: a low light level that simulates the light conditions of these crepuscular moths, and a high light level at which visual feedback should be more prominent. In both light levels, moths improved their ability to locate the nectary and feed from the flower. However, at the higher light levels, they took longer and were less effective in the feeding process. Why increased light should impair feeding performance remains an open question. These results also help illuminate how animal behaviour is impacted by increased light pollution in habitats near human populations.

In addition to the dual roles of mechanosensory and visual feedback, the role of odours in modulating visually guided behaviours was the focus of the third paper by Cheng & Frye [8]. This paper shows that responses to small objects in the visual field of flies elicit aversive behaviours; flies tend to steer away from small objects in the margins of their field of view. Cheng & Frye used a flight arena in which tethered flies, again *D. melanogaster*, freely rotated and performed steering manoeuvres, which were detected by both body rotation and wing-driven turns (via a wingbeat analyser). These experiments revealed that flies do indeed tend to steer and rotate away from small objects. This behaviour would of course be maladaptive if the fly needed to aim towards a food source in their visual field. Because relevant food sources have odours associated with them, the authors posited that the addition of a plume of odour (e.g. apple cider vinegar) would elicit frontal fixation of an object, which was indeed the case. Thus, flies showed a greater probability of frontal fixation (flying towards the object) and a lower probability of spinning and aversive steering. Taken together with Ruiz & Theobald's experiments [5], these data suggest interesting additional experiments that might reveal an odour-gated visual behaviour, depending on whether objects are presented in the dorsal versus ventral fields of the visual world.

Frontal fixation of visual images is part of gaze (head) stabilization, a key flight control behaviour that can reduce motion blur, particularly in fast aerial manoeuvres. This was the focus of the fourth paper, by Chatterjee *et al*. [9], which explored the interaction between mechanosensory information mediated by the antennal Johnston's organ and visual feedback in the oleander hawkmoth, *Daphnis nerii*. Their study highlights the critical aspects of sensory processing time—with fast mechanosensing occurring alongside slower visual image processing. These diverse sensory modalities, operating as negative feedback loops, have considerably different timescales [10], which likely results in emergent oscillatory dynamics. In the case of gaze stabilization, the authors showed that the head of the hawkmoth *D. nerii* undergoes lateral wobble (small-amplitude oscillations) [9]. A series of experimental manipulations of the mechanosensory antenna (e.g. clipping mass of the flagellum, gluing the Johnston's organ) revealed that the amplitude of head wobble increases when the mechanosensory information is reduced. Interestingly, light level changes (as also explored by Deora *et al*. [6]) may also influence the emergent dynamics, because lower light levels mean longer processing times. These data also suggest that the frequency of head wobble is lower under darker conditions. Thus, the enhanced head wobble may be an outcome of broken or delayed mechanosensory feedback acting within a larger reflex loop.

Visual feedback occurs across many wavelengths, and in a number of studies, polarized light plays an especially important role (e.g. [11]). The fifth paper, by Uemura *et al*. [12], explored how polarized light influences the behaviour of larval insects as they navigate terrestrial environments. They focussed on an intriguing emergent behaviour in which social caterpillars of two moth species, the European *Thaumetopoea pityocampa* and the Australian *Ochrogaster lunifer* (Lepidoptera: Notodontidae), walk single file, crawling head-to-tail as they navigate to feeding sites or pupation sites, laying down a silk trail along the way. Whereas chemosensory (e.g. pheromone) and mechanosensory input (tactile information between individuals) likely support this behaviour, the role of visual feedback is essential in reinforcing this group behaviour. As it turns out, the polarization of light detected by a novel visual organ identified by the authors plays a key role in the multisensory behaviour. Thus, orientation is maintained via feedback from multiple sensory inputs including odour and tactile inputs, as well as external environmental visual input.

## Data Availability

This article has no additional data.
